# Historical and future heat-related mortality in Portugal’s Alentejo region

**DOI:** 10.1186/s12889-024-21058-8

**Published:** 2024-12-20

**Authors:** Dora Neto, Miguel Bastos Araújo

**Affiliations:** 1 Rui Nabeiro Biodiversity Chair, MED – Mediterranean Institute for Agriculture, Environment and Development & CHANGE – Global Change and Sustainability Institute, Universidade de Évora, Largo dos Colegiais, Évora, 7004-516 Portugal; 2 Biogeography and Global Change Department, National Museum of Natural Sciences, CSIC, C/ Jose Gutierrez Abascal, 2, Madrid, 28006 Spain

**Keywords:** Heat-related mortality, Short-term effects, Climate change, Alentejo

## Abstract

**Background:**

The increased severity of extreme weather and anticipated climate change has intensified heat stress-related mortality worldwide. This study examines the historical short-term effects of heat on mortality in Alentejo, Portugal’s warmest region, and projects it up to the end of the century.

**Methods:**

Using data from 1980 to 2015 during warm seasons (May-September), the association between daily mortality by all-causes and mean temperature was examined following a case time series design, applied at both regional and subregional scales. Projections for daily temperatures were obtained from regional climate models and greenhouse gas emission scenarios (RCP4.5, RCP8.5). We also examined temporal shifts in mortality considering potential long-term and seasonal adaptative responses to heat. We then quantified the yearly effects of heat by calculating absolute and relative excess mortality from 1980 to 2015, specifically during the heatwave of 2003 (July 27 to August 15), and in future projections at 20-year intervals through 2100.

**Results:**

The analysis revealed a significant rise in mortality risk at temperatures exceeding a minimum mortality temperature (MMT) of 19.0 °C, with an exponential trend and delayed effects lasting up to 5 days. The risk increased by 413% at the maximum extreme temperature of 36.6 °C. From 1980 to 2015, 2.32% of total deaths, equating to over 5,296 deaths, were heat-associated. No significant shifts over time were noted in the population’s response to heat. Future projections, without adaptation and demographic changes, show a potential increase in mortality by 15.88% under a “no mitigation policy” scenario by 2100, while mitigation measures could limit the rise to 6.61%.

**Conclusion:**

Results underscore the urgent need for protective health policies to reduce regional population vulnerability and prevent premature heat-related deaths across the century.

**Supplementary Information:**

The online version contains supplementary material available at 10.1186/s12889-024-21058-8.

## Background

Human exposure to temperatures beyond the optimal range, either excessive heat or cold, increases mortality and morbidity rates. With global warming amplifying the intensity, frequency, and duration of extreme events [1, 2], the resulting mortality is a burgeoning global public health concern [2–7]. The effects of temperature changes on human health are contingent upon a combination of intrinsic (i.e., physiological acclimatization) and extrinsic processes (e.g., socioeconomic, infrastructural, technological, among others). Together, they define the overall capacity of human populations to adapt to their climate [3, 8–10]. Intrinsic and extrinsic processes manifest themselves differently depending on their location and temporal circumstances. Consequently, thermal tolerances and risks vary across countries and regions, and even between regions within the same country [3, 8, 9, 11–14]. All else being equal, differences on thermal susceptibility are also found by population characteristics, such as age and sex [3, 8], and/or pre-existent clinical conditions, such as cardiovascular and respiratory diseases, among others [3, 10, 15].

The Mediterranean region, encompassing the Alentejo region of Portugal, is highly susceptible to climate change. According to the latest report from the Mediterranean Experts on Climate and Environmental Change (MedECC), annual mean temperatures have already risen 1.5 °C beyond pre-industrial levels, marking a rate 20% faster than the global average [16]. Future projections from the Intergovernmental Panel on Climate Change’s (IPCC) Sixth Assessment Report (AR6), based on ensemble means from the two latest phases of the Coupled Model Intercomparison Project (CMIP5 and CMIP6), suggest that summer warming in the Mediterranean could exceed global annual warming by 40–50%. This warming is expected to be accompanied by more frequent and intense heatwaves [2]. Under the most extreme greenhouse gas emissions scenario (RCP8.5), by century’s end, most of Alentejo is projected to endure, on average, 120 to 140 hot days annually (days exceeding 30 °C), a marked leap from the 60–80 days recorded between 1971 and 2000. Furthermore, the annual average of heatwaves (number of days with at least six consecutive days when daily maximum temperature exceeds the 90th percentile, obtained for the calendar day using a 5-day moving window) is projected to increase from 0 to 20 to 140–180 days by the end of the century [4].

In response to the devastating 2003 heatwave in Europe, the Portuguese General Directorate for Health (“Direção Geral da Saúde”) established a seasonal contingency plan [17] to prevent the most adverse effects of heat. This plan mandates Regional Health Authorities to orchestrate responses at various levels, leveraging national to local data streams, including meteorological forecasts. Criteria for activating heat health alerts, such as critical temperature thresholds and the íCARO-alert-index [18], are assessed comprehensively. Recommendations for issuing alerts are calibrated to higher temperatures later in the season (July-September) due to assumptions of differential regional vulnerabilities [17]. The plan also encourages tailoring these criteria to regional specifics, underscoring the need for localised research, given that the precision of risk indicators markedly influences their capacity to signal health hazards, which vary by location and timing. Heat action plans and warning systems must be underpinned by area-specific climatic, environmental, and health data to accurately reflect local risk associations [3, 5–7, 19, 20].

While environmental epidemiological inquiries into health implications of warmer temperatures often adopt a descriptive methodology, comparing specific heat wave incidents to homologous time periods [3, 21], some simplify the modelling of exposure-response associations [18, 22]. Yet temperature effects are continuous and characteristically non-linear and with lagged responses [20, 23, 24], generally persisting over a few days for warmer temperatures. Neglecting these dependencies can introduce biases in estimates and their confidence intervals [20, 24]. Additionally, while conventional time series analyses that amalgamate data over extensive areas or populations can be informative, they may mask local spatiotemporal variations, thereby potentially misrepresenting exposure risks [25, 26].

In this study, using multiple fine-scale (municipal) time series data from 1980 to 2015 in Alentejo, we investigate the short-term effects of heat on mortality at the regional and subregional scale, exploring whether the association is consistent across the territories and whether there is evidence of regional temporal shifts in the effects. We also quantify the mortality burden for the past and future, extending projections until the end of the 21st century. By doing so, we equip regional decision-makers and stakeholders with crucial insights to refine or devise efficacious interventions to curtail heat-related mortality now and in the anticipation of future climatic shifts.

## Methods

To elucidate the relationship between heat and mortality risk in Alentejo, we employed a case-time series approach using a conditional Poisson model with a Distributed Lag Non-linear Model (DLNM). The model was fitted separately for each of Alentejo’s four subregions to investigate geographical differences that might arise because of multiple factors (e.g., health, climate, demographics, socioeconomics). We also examined temporal shifts in Alentejo’s mortality, considering potential long-term and seasonal adaptive responses to heat. We then quantified both period-wide and annual regional effects of heat by calculating absolute and relative excess mortality from 1980 to 2015, with a particular focus on the heatwave of 2003 (July 27 to August 15). Additionally, we assessed past overall impacts at the subregional level and analysed regional and subregional impacts through historical and future projections at 20-year intervals through 2100.

### Study locations and datasets

The study encompassed the Alentejo (*n* = 47 municipalities) and its four subregions Figure S1): Alentejo Litoral (*n* = 5), Alto Alentejo (*n* = 15), Alentejo Central (*n* = 14) and Baixo Alentejo (*n* = 13), each assessed separately. Daily mortality (all causes) and mean air temperature were collected for each municipality from 1980 to 2015. Impact projections used high-resolution ensemble simulations (1980–2100) that incorporated daily temperature data from five climate models across two greenhouse gas emission scenarios. Analyses focused on heat effects from May 1st to September 30th (extended summer).

The Portuguese National Institute of Statistics (INE) supplied mortality data, adhering to strict confidentiality standards. Deaths were categorised using the ICD-9 (International Classification of Diseases; up to 2002) and ICD-10 (after 2002) revision. For increased accuracy we excluded deaths related to mental health and other external causes. Specifically, cases coded in Chapters V (Mental and behavioral disorders, ICD-9/10), XVII (Injury and poisoning, ICD-9), XIX (Injury, poisoning and certain other consequences of external causes, ICD-10) and XX (External causes of morbidity and mortality, ICD-10). We aggregated data by day, municipality, age, and gender into a daily all-cause mortality series.

Mean daily temperature was calculated by averaging minimum and maximum temperatures using the Iberia01 observational gridded dataset (1971–2015, 0.10º or ~ 10 km resolution), obtained from 275 weather stations for temperature across the Iberian Peninsula [27], including 11 located within the study area. Similarly, projected daily mean temperatures were averaged from modelled daily extremes, drawing from five simulation sets in the EURO-CORDEX domain [28] of the Coordinated Regional Downscaling Experiment (CORDEX) initiative [29]. These sets, originating from five regional circulation models (RCM), at horizontal resolution of 0.11° (~ 12.5 km), driven by several global circulation models (GCM) used in the IPCC’s AR5, spanned historical (1950–2005) and future (2006–2100) periods under two Representative Concentration Pathways (RCP4.5 and RCP8.5). RCP4.5 represents a moderate mitigation scenario in which pollutant emissions start decreasing after 2040, while RCP8.5 underlines a “no-climate-policy” scenario where emissions keep rising [30]. The list of RCM-GCM simulations, available at the Earth System Grid Federation (ESGF) data portal, is provided in Table S1.

To align RCMs and observational-gridded data, we used a parametric type of quantile-mapping approach that preserves the climate change signal. This method was developed by Hempel et al. [31] as part of the first Inter-Sectoral Impact Model Intercomparison Project (ISI-MIP). The final climatic time series datasets consisted of daily mean temperatures per municipality, which were computed as weighted-area averages of the grid cell values in each location.

Analyses were conducted using R (version 4.2.2) [32] with the primary packages “ncdf4” [33], “terra” [34] and “sf” [35] for spatially analysis and the “fhempel” function [36] for bias adjustments.

### Statistical analysis

#### Temperature-mortality relationships

Using a case time-series design [25, 26], which parallels a case-crossover approach [37], we assessed the short-term mortality risks linked with seasonal temperature fluctuations (time varying), within Alentejo’s municipalities from 1980 to 2015. Briefly, the conditional Poisson regression model [37] was applied to fit temperature and mortality data, allowing for stratified baseline mortality risks, while accounting for long-term and seasonal trends at both local and regional levels [26]. The association between temperature and mortality was characterised using a DLNM accounting for nonlinear and delayed effects [23], with a lag window of 0–10 days to understand any postponed effects and potential short-term mortality displacement [13, 20, 38]. Further statistical details and technical aspects of the modelling framework are provided in the supplementary material (p. 2).

The relationship between temperature and mortality was expressed as an overall cumulative relative risk (RR) with 95% confidence intervals (95% CIs). This metric of RR was viewed against the optimum or minimum mortality temperature (MMT), the exposure with the lowest associated risk, which is often considered an indicator of long-term adaptation to local climate [10, 39]. We plotted temperature and lag effects for select days (0, 3, 5, 7, and 10) and temperature intensities (50th, 75th, 95th, 99th percentiles, and maximum).

Lastly, in a detailed subregional and temporal examination, we used the same methodological framework and regression model settings applied across the whole Alentejo to study the subsets of data. To assess the overall cumulative exposure-response relationships across the subregions, we utilized the Alentejo-specific MMT as a standard reference. Although this method accounts for spatial variations in temperature sensitivities and allows for inter-area risk comparisons, it does not consider potential disparities in optimal temperature thresholds. To address this, and investigate potential long-term and seasonal adaptative responses to heat, we conducted a separate temporal analysis within Alentejo, segmenting the timeline into two intervals, 1980–1997 and 1998–2015, and further stratifying by early (May-June) and peak (July-September) summer periods. We evaluated variations in heat vulnerability at the 50th, 75th, 95th, and 99th temperature percentiles of Alentejo’s distribution. The differential susceptibilities to heat were statistically verified using the Z-statistic [40].

#### Sensitivity analysis

To test the robustness of our regional model, we conducted a sensitivity analysis (see Figure S2). The following modifications were made:

1. Lag period adjustment: we tested both shorter (up to 5 days) and extended (up to 14 days) periods.
2. Knot placement variations: The positions of the knots in the natural cubic spline were adjusted based on different combinations of temperature percentiles - (i) 50th, 75th, and 90th, (ii) 50th and 75th, and (iii) 75th and 90th percentiles.
3. Stratum modification: We expanded the stratum specification by removing the monthly component.

In our temporal analysis, we omitted years marked by anomalous heat-related mortality spikes, as exemplified by 2003, or as detected in the annual impact assessments specific to the Alentejo region. This approach ensured that our results were not skewed by atypical data points, providing a more accurate reflection of the general relationship between heat and mortality (see below).

#### Assessment on heat-related mortality impact

Using Gasparrini and Leone’s method [41], we assessed heat’s contribution to mortality. Through a forward estimation technique, we measured the mortality impact as the attributable fraction (AF) and number (AN). The AF, considering a 10-day lag, captures the accumulated effects from current exposures compared to the reference temperature (regional MMT). From this estimation and the average daily deaths across the lag window, we derived the daily AN. We restricted the heat impact calculations to exposure intensities above the reference value. The sum of the daily AN within a given time range defined the overall absolute excess heat-related mortality (prevalence), and its ratio with the total number of deaths defined the overall relative excess heat-related mortality (%).

For a comprehensive understanding, both overall AN and AF were evaluated separately for the Alentejo and its subregions over the period 1980–2015 and projected 20-year intervals up to 2100. Furthermore, Alentejo’s yearly impacts were analysed with special attention to the severe 2003 heatwave from July 27 to August 15. For the study period analysis (1980–2015), we used the observed temperature-mortality data and exposure-response curves, with reduced lag dimension, specific to each area. For future projections, we adapted Vicedo-Cabrera et al.’s method [36]. We constructed projected daily death series from observed counts, replicating and aligning average counts per seasonal day (Figure S3) with the projected climate data’s duration. The exposure-response curve’s natural cubic spline function of each area was log-linearly extrapolated to accommodate temperature values and effects beyond the current period. Given data from various climate models and scenarios, our results are conveyed as ensemble averages with point estimates reflecting the mean across the RCM-RCP groups [36, 42].

Finally, we incorporated an uncertainty assessment. Using Monte Carlo simulations (*n* = 1000) and assuming a multivariate normal distribution of the regression model’s coefficients, empirical 95% confidence intervals (eCIs) were computed for the observed point estimates [41] by geographical area. This method was extended to the impact projections, considering variations from the different climate models and scenarios, as the outcomes represent ensemble averages [36].

The “gnm” [43] and “dlnm” [44] packages were used to fit the regression models and distributed lag nonlinear models, respectively.

## Results

Between 1980 and 2015, the Alentejo region documented 227,835 all-cause deaths. Of this total, 36.75% (or 83,701) occurred during the May to September period. Most of these seasonal deaths, amounting to 83.1%, were among individuals aged above 65 years old (Table 1).

**Table 1:** Descriptive statistics of the daily mean temperature and mortality for Alentejo and subregions. Parameters were determined based on 12 months of the year and/or seasonal months (MJJAS) from 1980 to 2015

Daily mean temperature [ºC]					Daily mortality [*n*]											
	Mean		Range	IQR		Sum					Mean			Range		IQR
						All ages			(+ 65 years)		All ages					
Subregion/Region	12 months	MJJAS	MJJAS			12 months ^(1)^	**MJJAS** ^(2)^	**%** ^(2/1)^	MJJAS (n)^(3)^	% ^(3/2)^	12 months	MJJAS	MJJAS			
Alentejo Central	16.69	22.18	28.76	5.35		65,404	24,063	36.79	19,975	83.01	0.36	0.31	6		0	
Alentejo Litoral	15.88	20.28	24.44	4.04		38,159	14,073	36.88	11,448	81.35	0.58	0.51	6		1	
Alto Alentejo	16.39	22.07	29.26	5.68		59,289	21,708	36.61	18,432	84.91	0.30	0.26	5		0	
Baixo Alentejo	16.98	22.30	27.81	4.97		64,983	23,857	36.71	19,842	83.17	0.38	0.33	7		1	
Alentejo	16.59	21.97	30.02	5.29		227,835	83,701	36.75	69,697	83.11	0.37	0.32	7		1	

MJJAS = May, June, July, August, and September; IQR = interquartile range (Q3-Q1)

The mean daily death count was low across all subregions, leading to many seasonal days with no recorded deaths at the municipal level. Alentejo Litoral experienced the least seasonal mortality with 14,073 deaths but had the highest daily mean mortality rate by municipality (mean = 0.51). Alentejo Central recorded the highest overall mortality, with 24,063 deaths (mean = 0.31), followed closely by Baixo Alentejo with 23,857 (mean = 0.33) and Alto Alentejo with 21,708 (mean = 0.26). Despite generally stable mortality rates, Alentejo Litoral and Baixo Alentejo showed greater day-to-day fluctuations in mortality (IQR = 1, Table 1; Figure S3).

The annual and seasonal average daily mean temperature in Alentejo was 16.59ºC and 21.97ºC (IQR = 5.29ºC), respectively. There were marked annual and seasonal variances, particularly between coastal and inland areas. Municipalities on Alentejo Litoral usually had daily temperatures about 2ºC lower than those in inland subregions (Table 1, Figure S4).

**Fig. 1:**
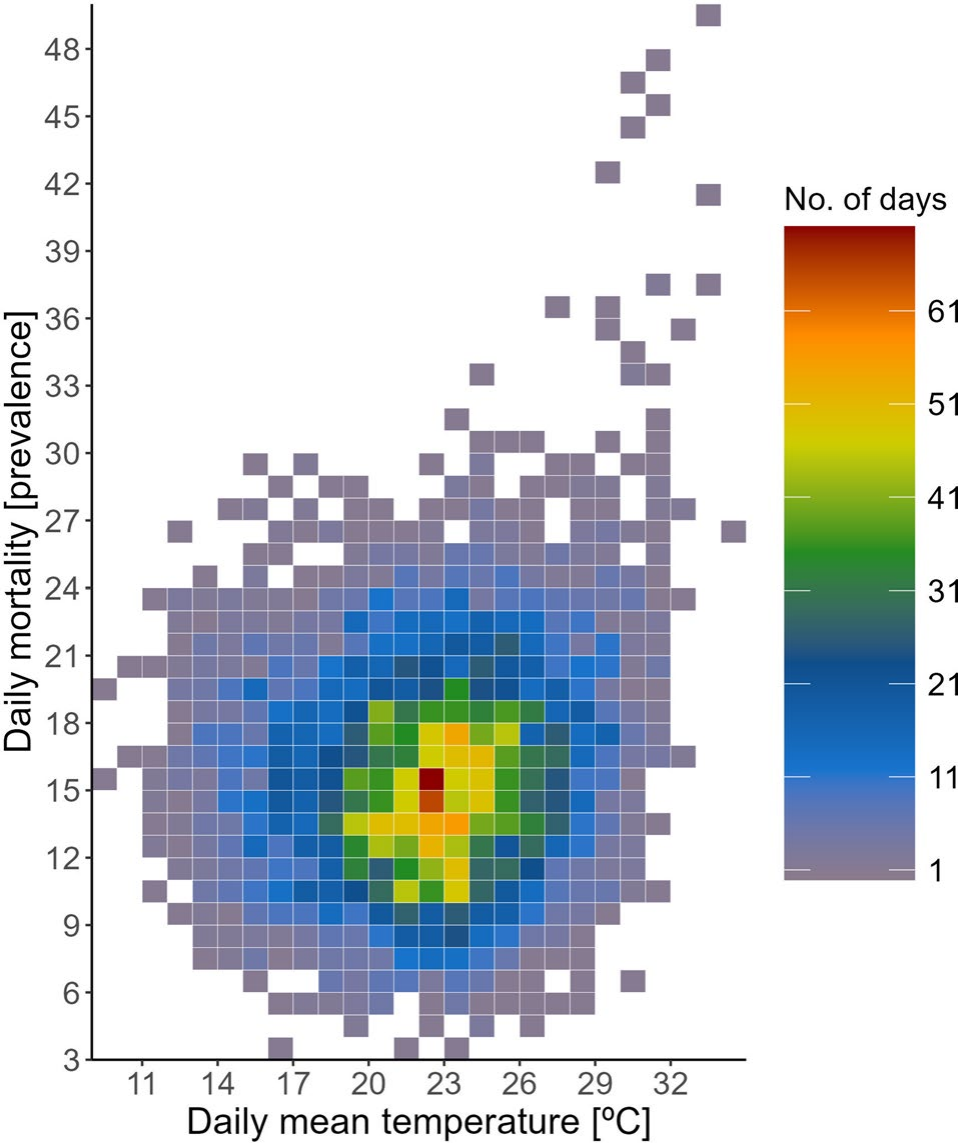
Seasonal distribution (May-September) of observed daily mortality across Alentejo´s municipalities, grouped by day, illustrating the relationship with the average daily temperature gradient. The heatmap indicates the accumulated days across the 1980–2015 period associated with each combination (X_x_, Y_y_)

Most of the season’s days in Alentejo with increased mortality prevalence clustered around mean temperatures between 19ºC to 26ºC. However, a pronounced surge in mortality was observed on days when temperatures exceed this range, as indicated by the right tail of the distribution shown in Fig. 1. This trend underscores the heightened risk of heat-related deaths on particularly warm days, beyond the region’s typical temperature norms.

**Fig. 2:**
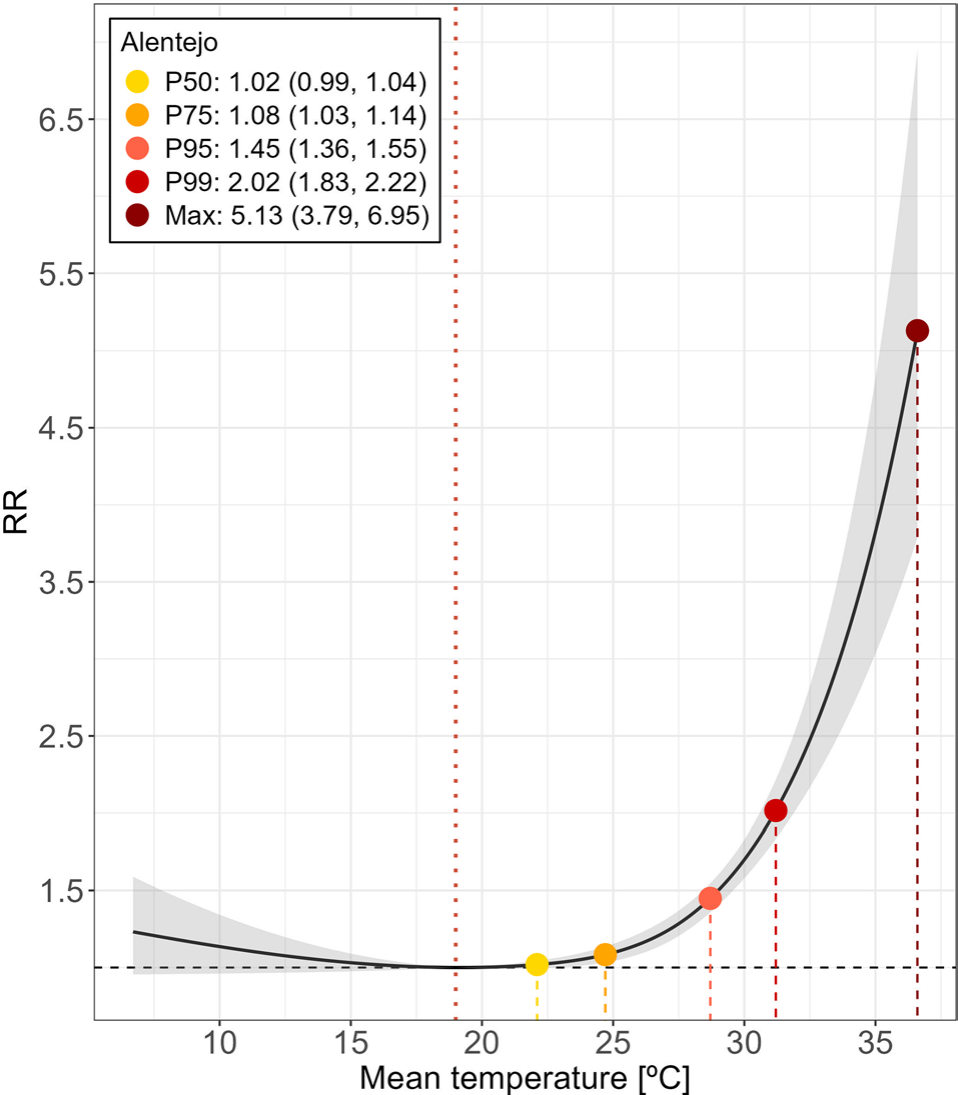
Overall association between mean daily temperature and mortality for Alentejo (1980–2015). Black curve represents the cumulative relative risk (RR) over the maximum lag period (10 days) using the reference at 19.0 °C (MMT, orange dashed vertical line). Effects depicted at 50th (P50: 22.1 °C), 75th (P75: 24.7 °C), 95th (P95: 28.7 °C), 99th (P99: 31.2 °C) and maximum extreme (36.6 °C) of temperature distribution. Shaded area represents 95% confidence intervals

The MMT for Alentejo is 19.0 °C, and it is at this inflection point where the cumulative risk of mortality begins to climb sharply, as depicted in Fig. 2 and anticipated in Fig. 1. The relationship follows a J-shaped curve, with the risk of death increasing exponentially. This rise ranges from a 2% increase in risk (RR = 1.02, 95% CI: 0.99–1.04) at the 50th percentile of temperature, 22.1 °C, to a substantial 413% increase (RR = 5.13, 95% CI: 3.79–6.95) at extreme temperatures, such as 36.6 °C.

Temperature’s most pronounced impact on mortality was immediate, occurring on the same day of exposure (Lag 0). At this lag, the risk increment ranged from 4% (RR = 1.04, 95% CI: 1.03–1.05) at a median temperature of 22.1 °C (50th percentile) to 62% (RR = 1.62, 95% CI: 1.44–1.83) at the extreme temperature of 36.6 °C, when compared to the MMT (Figure S5, left panel). Additionally, the analysis revealed the presence of delayed adverse effects, whose duration and severity increased with the intensity of the temperature exposure. At the highest temperature extreme, the elevated risk remained significant for up to 5 days post-exposure (Lag = 5; RR = 1.07, 95% CI: 1.02–1.12). Our findings also suggest the absence of short-term mortality displacement, as no negative reduction in mortality was observed following the initial peak within the lag window. This indicates sustained effects rather than a temporary shift in mortality timing (Figure S5, right panel).

Sensitivity analyses confirmed the robustness of the cumulative exposure-response relationship, particularly in its overall configuration, as shown in Figure S2. Modifying the maximum lag duration led to a lowered estimated MMT of 13ºC, though with reduced precision in the upper range of the temperature curve. A similar trend was observed when increasing the number of knots across the temperature spectrum or adjusting the case and time strata observation counts, leading to slightly increased uncertainty at moderate temperatures. Adjusting knot placement alone preserved the estimated MMT but increased uncertainty for temperature fluctuations at the 75th and 90th percentiles, while slightly reducing it at the 50th and 75th percentiles. Despite these variations, the average risk levels remained relatively consistent across different model specifications, underscoring the stability and reliability of the results.

**Fig. 3:**
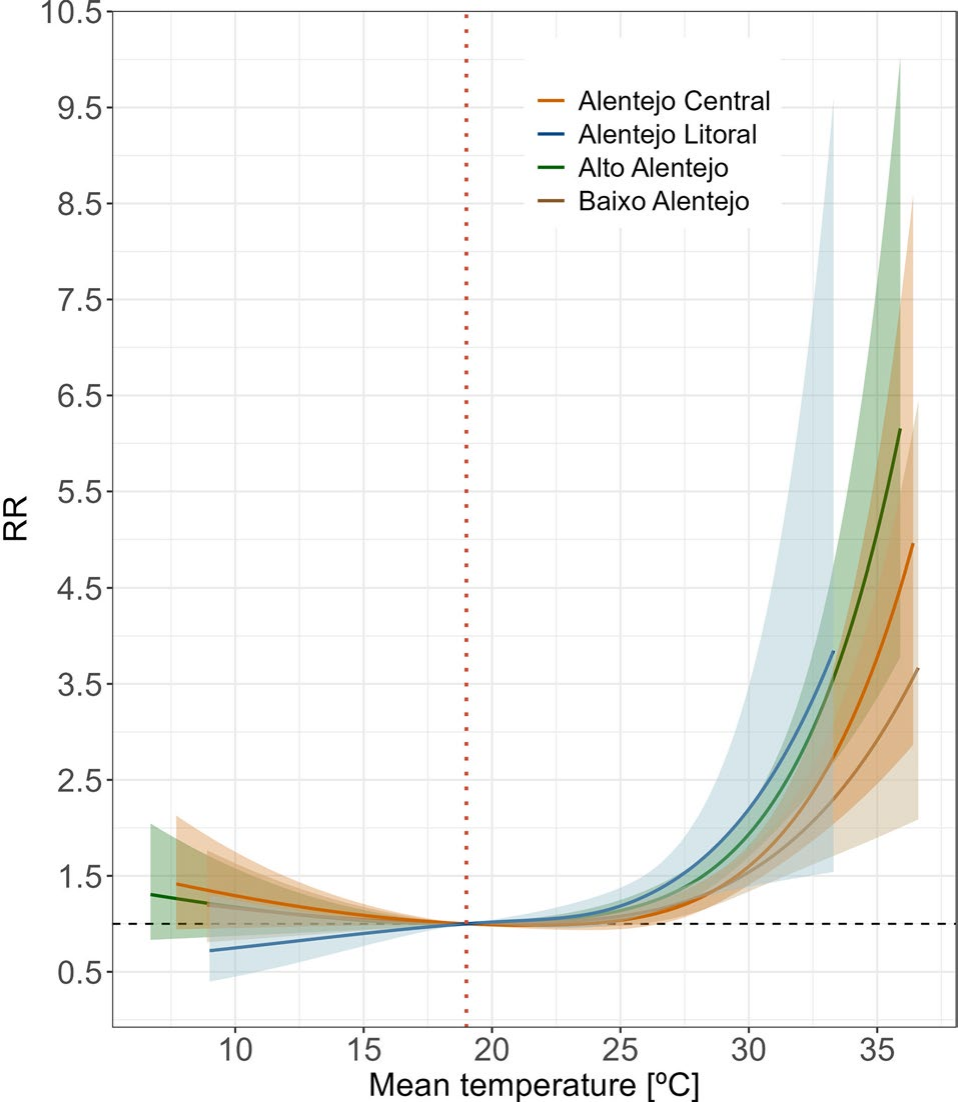
Overall association between mean daily temperature and mortality for Alentejo subregions (1980–2015). Curves represent the cumulative relative risk (RR) over the maximum lag period (10 days) regarding the regional reference temperature (MMT = 19.0 °C, orange dashed line). Shaded areas represent 95% confidence intervals

Subregional analyses, using the regional MMT as a benchmark, consistently exhibited an exponential increase in RR, paralleling the general trend observed for the entire Alentejo region (Fig. 3). There was a minor variation in risk among subregions at comparable levels of exposure, with the cooler Alentejo Litoral showing somewhat heightened effects and the warmer southern Baixo Alentejo exhibiting lower effects. Nonetheless, the overlapping 95% CIs suggest that it is not possible to make a definitive distinction between the subregions’ heat sensitivities, possibly due to limited statistical power from the smaller number of cases analysed.

**Fig. 4:**
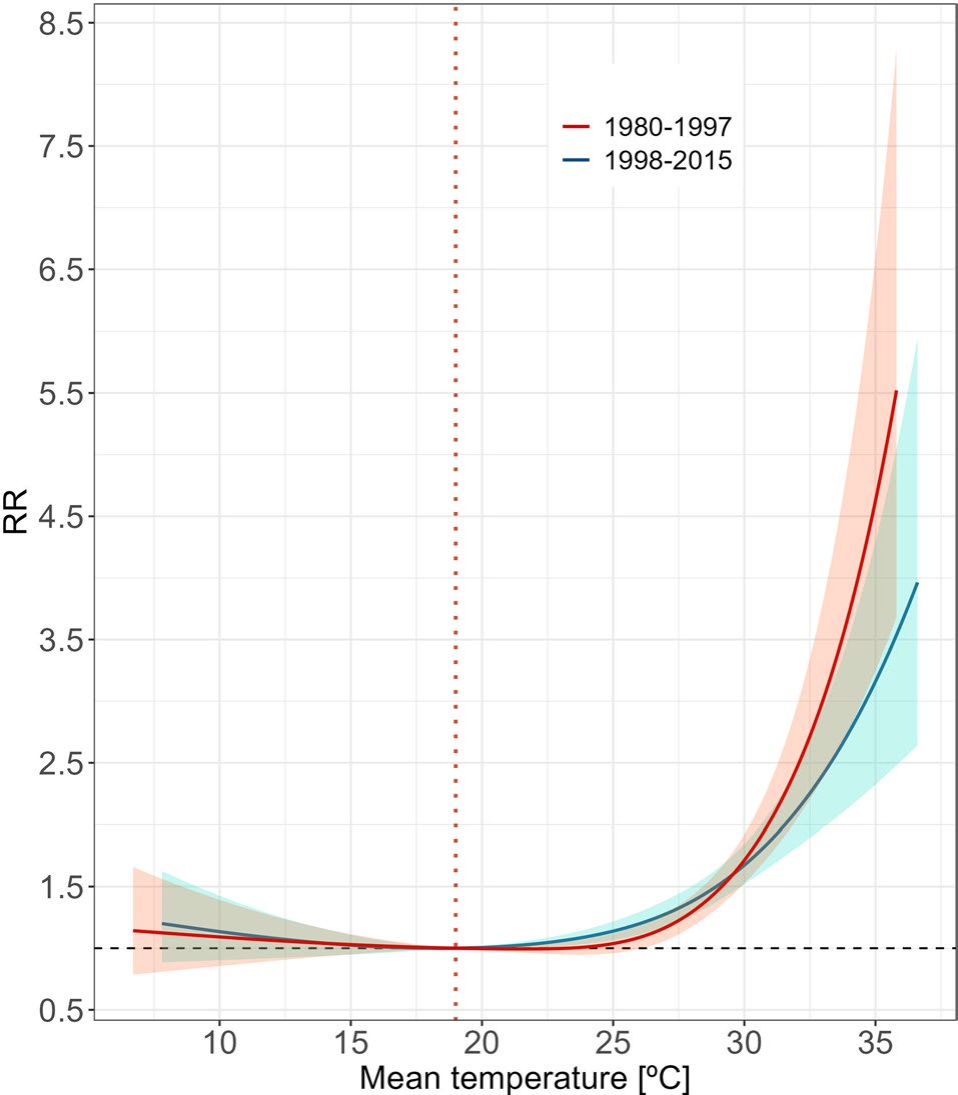
Overall association between mean daily temperature and mortality for Alentejo by time subperiods. Curves represent the cumulative relative risk (RR) over the maximum lag period (10 days) regarding the reference temperature (MMT = 19.0 °C, orange dashed line). Shaded areas represent 95% confidence intervals

The temporal analysis confirmed the MMT for Alentejo at 19ºC, aligning with the broader regional results. During the 1998–2015 subperiod, significant health effects emerged at the 75th percentile of the temperature distribution, corresponding to 24.7ºC (Fig. 4, Table S2). This pattern was consistent across both subperiods at the 95th (28.7ºC) and 99th (31.2ºC) percentiles, indicating increased risk at these higher temperatures, especially during the earlier period. Despite these findings, no statistically significant difference was found in the temperature thresholds between the two subperiods (*p*-value > 0.05, Table S2).

**Fig. 5:**
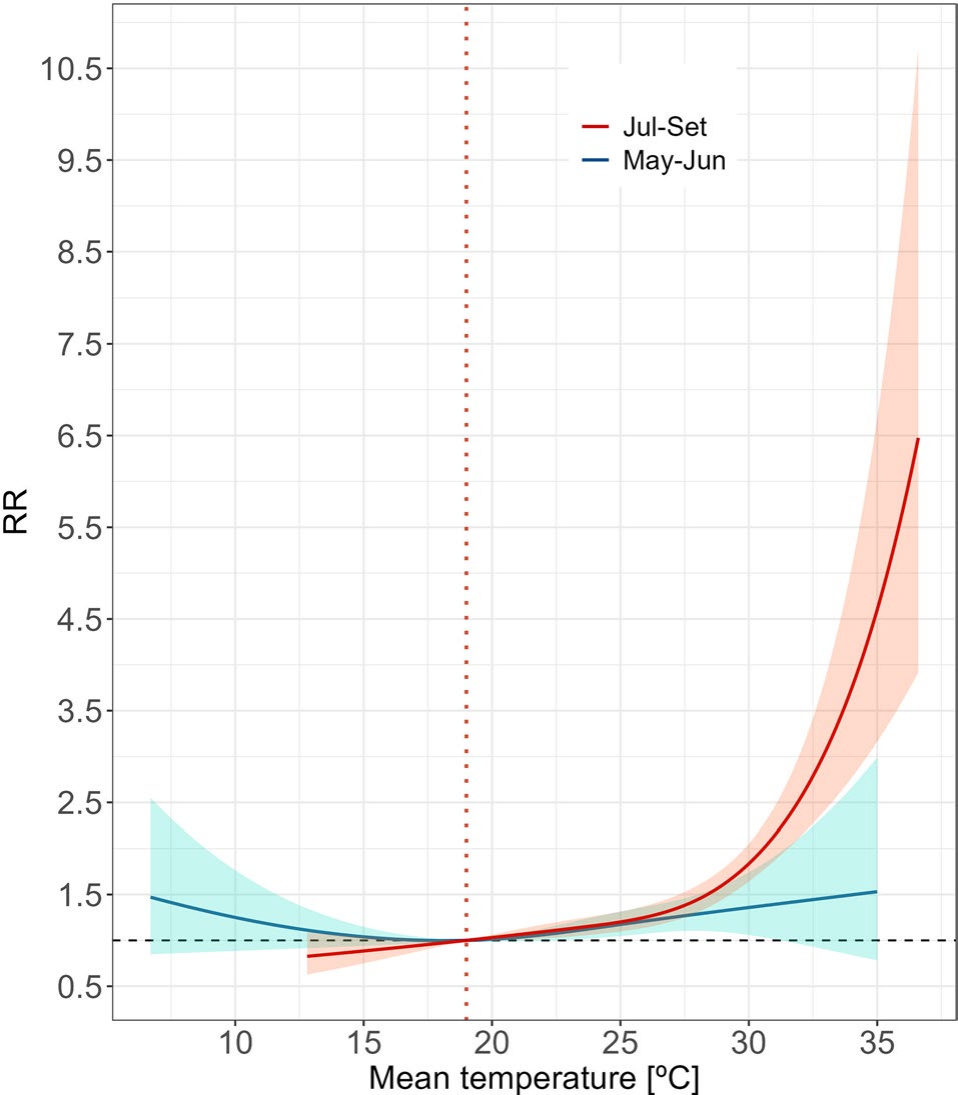
Overall association between mean daily temperature and mortality for Alentejo by seasonal subperiods. Curves represent the cumulative relative risk (RR) over the maximum lag period (10 days) regarding the reference temperature (MMT = 19.0 °C, orange dashed line). Shaded areas represent 95% confidence intervals

During the summer season, we observed an increase in mortality risks during the latter part (July-September) (Fig. 5, Table S3). While statistical analyses did not reveal significant differences between the early (May-June) and late summer periods (*p*-values > 0.05, Table S3), this lack of distinctions - also noted in the long-term analysis - may be due to limited statistical power from the use of non-overlapping time divisions.

It is also important to note that omitting years with atypically high mortality rates, such as 1981, 1991, 2003, 2006, and 2010, resulted in only minor changes to the risk estimates, primarily noticeable at the extreme temperature levels (Tables S1-S2). This suggests that the observed temporal relationships between temperature and mortality are not solely driven by these outlier years, but rather reflect a consistent pattern across the entire study period.

Taken together, our results show no substantial evidence of decreased heat tolerance or an adaptative response to high temperatures among the populations of Alentejo, whether considered on a short-term scale (as indicated by within-season variation) or over a longer temporal framework. However, as noted, the limited statistical power may have partially hindered our ability to detect more subtle variations in heat sensitivity.

**Fig. 6:**
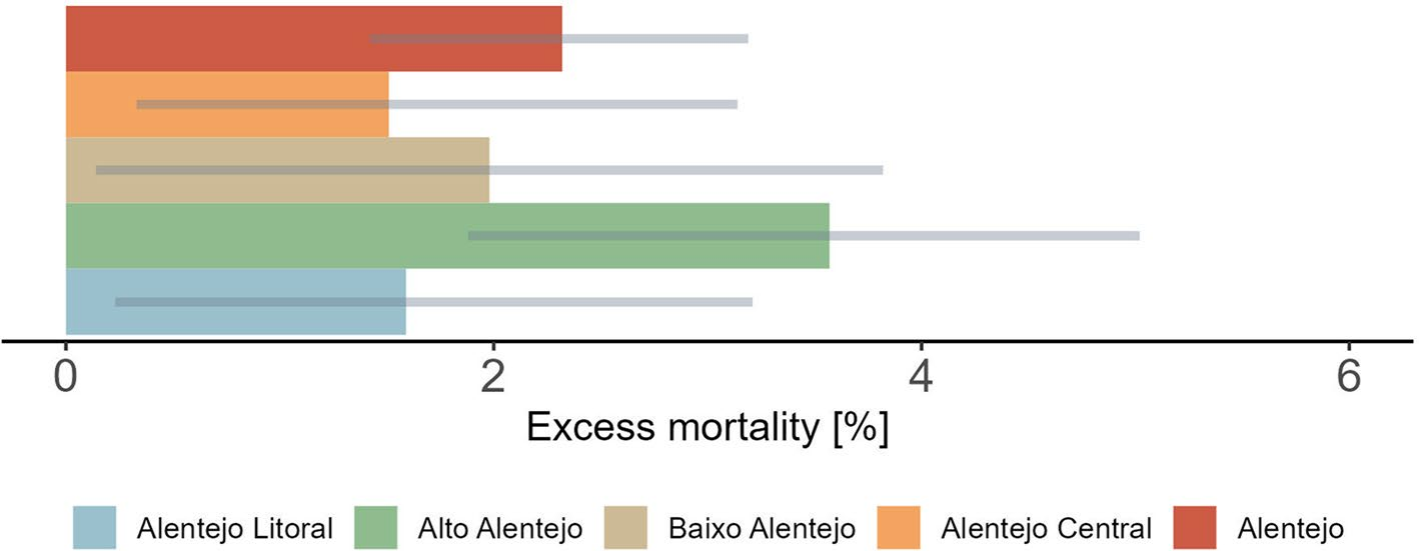
Overall relative excess mortality (%) associated with heat for Alentejo and subregions from 1980–2015. Impact estimates are based on comparisons with the regional MMT (19ºC), considering all the exposure intensities above this threshold. Grey lines represent empirical 95% confidence intervals

The analysis of heat-related impacts, based on data from 1980 to 2015 and considering temperatures above the regional optimum (MMT = 19ºC), indicates that 2.32% of all deaths in Alentejo were attributable to heat (Fig. 6). This corresponds to an estimated 5,296.4 deaths, with a 95% eCI ranging from 3,435.72 to 7,211.27 (Table S4).

**Fig. 7:**
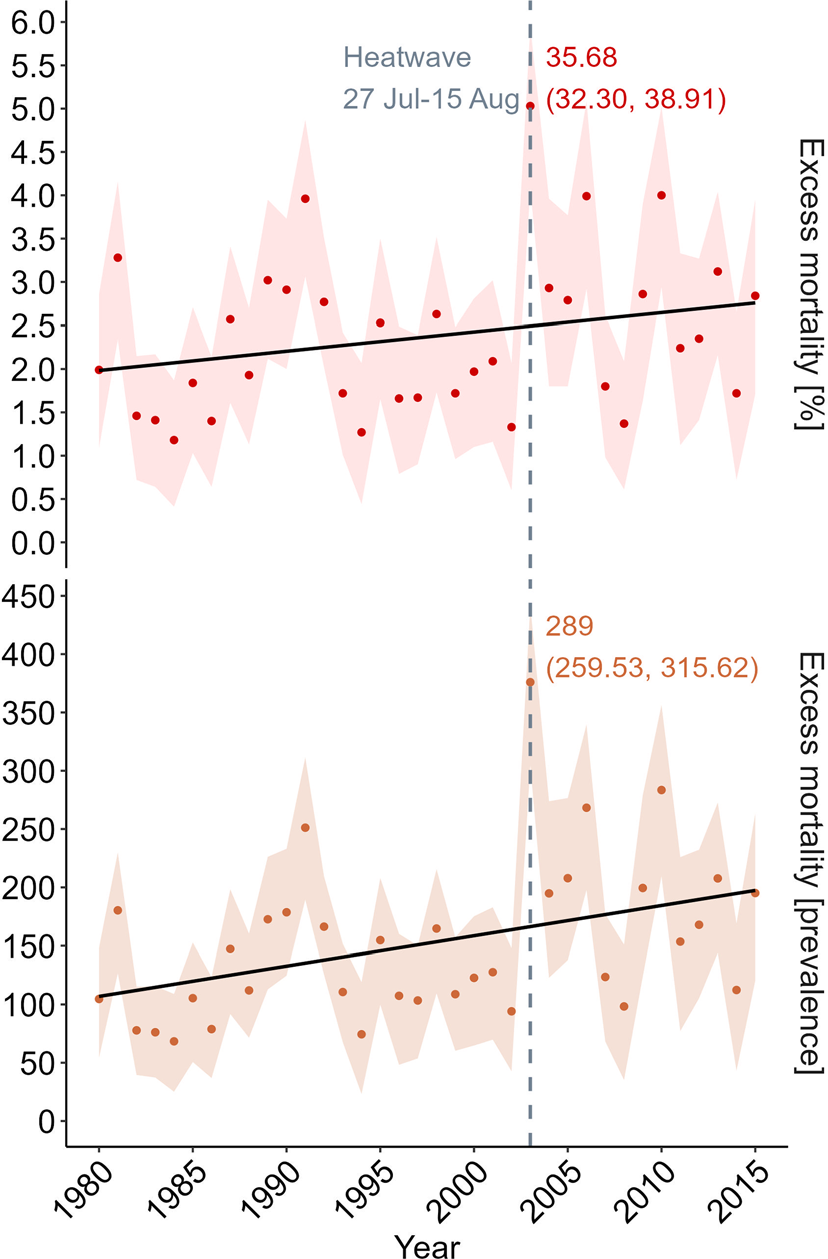
Temporal trajectory of annual excess mortality associated with heat in Alentejo from 1980 to 2015. Linear fit from linear regression model. Dashed line sets for the heatwave of 2003 (July 27 - August 15). Measures of overall absolute (prevalence) and relative (%) excess are in comparison with the MMT (19ºC) and considering all the exposure intensities above this threshold. Shaded areas represent empirical 95% confidence intervals

The annual seasonal impact assessment revealed a steady increase in heat-related mortality in Alentejo throughout the study period (Fig. 7). The severe heatwave of 2003, lasting from July 27 to August 15, resulted in a substantial overall excess of 289 deaths (95% eCI: 259.53–315.62) within the region. This equates to 35.68% (95% eCI: 32.30 − 38.91%) of all deaths during the heatwave period, representing most of the seasonal mortality attributed to warm weather in 2003, which totalised 375.93 deaths (95% eCI: 304.65–442.09).

When comparing subregions, Alto Alentejo exhibited the highest impact, with an overall estimate of 3.57% (95% eCI:1.88 − 5.02%), corresponding to 2,114.44 deaths (95% eCI: 1,227.47–3,006.92) (Table S4). However, in line with the RR uncertainty estimates (Fig. 3), there was a significant statistical overlap between the subregions (Fig. 6), which does not necessarily rule out the presence of true differences.

**Fig. 8:**
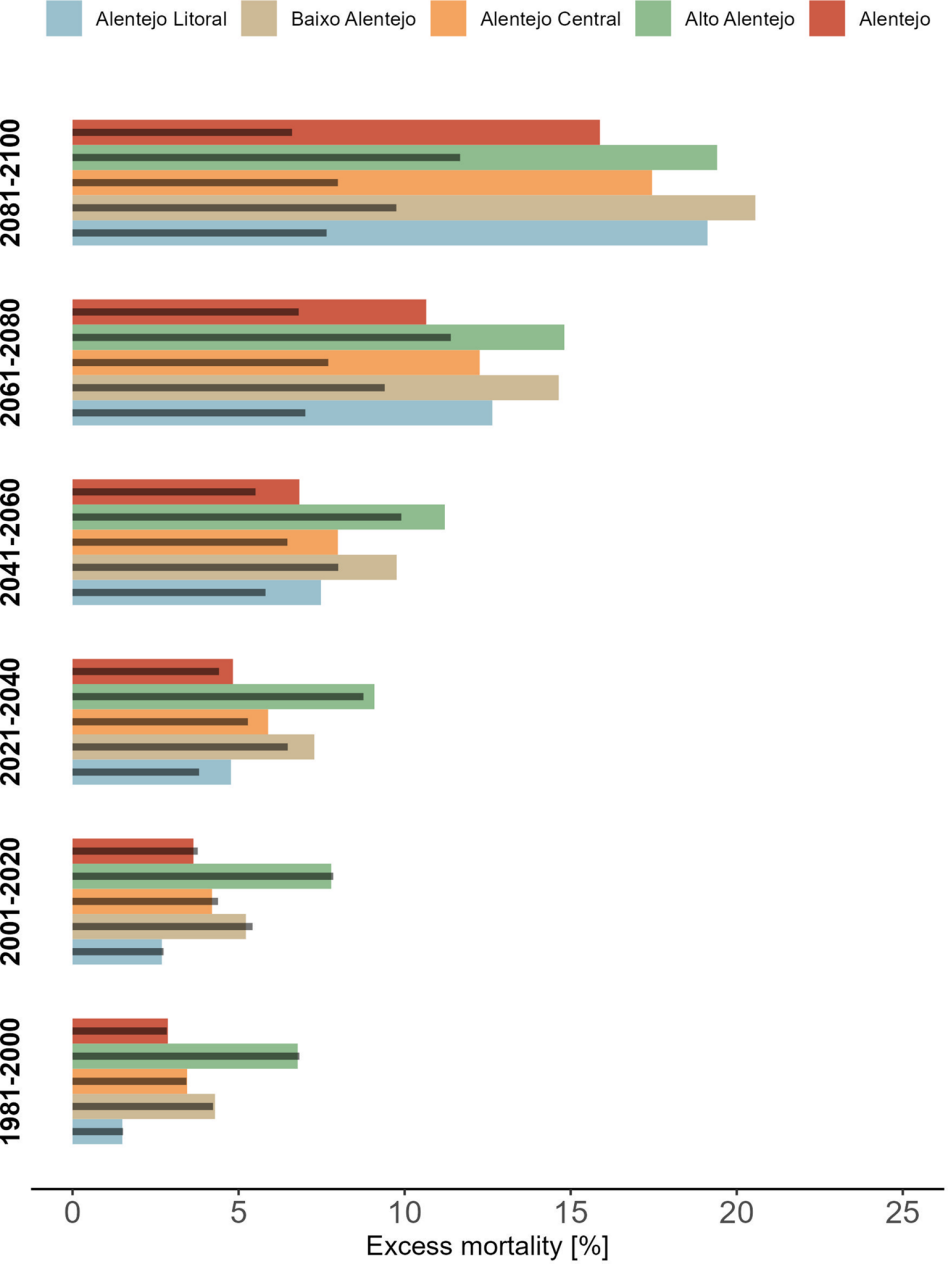
Projected overall relative excess mortality (%) associated with heat for Alentejo and its subregions from 1981–2100. Coloured and dark grey bars represent RCP8.5 and RCP4.5 scenarios, respectively. Impact estimates are based on comparisons with the regional MMT (19ºC), considering all the exposure intensities above this threshold

Future projections for Alentejo indicate that without necessary climate change adaptations and assuming unchanged demographics, heat-related deaths could rise from 3.64% (95% eCI: 0.31 − 7.61%) to 15.88% (95% eCI: 8.20 − 22.99%) between 2001 and 2020 and 2081–2100 if no mitigation policies are implemented (RCP8.5) (Fig. 8, Table S5). However, with mitigation measures (RCP4.5), the anticipated rise is more modest, resulting in a relative excess of 6.61% (95% eCI: 1.62 − 12.33%) by the end of the century. Both emission pathways show similar heat-related impacts up to 2040, and these qualitative patterns are consistent across the subregions.

## Discussion

This study provides a comprehensive assessment of past and future heat-related mortality in Portugal’s Alentejo region. Using a robust case time series approach, we present the first analysis of mortality risks and impacts at both regional and subregional levels, accounting for fine-scale epidemiological variations across territories. This approach minimizes exposure misclassification and reduces bias. In addition, by capturing the complex non-linear and delayed effects of temperature exposure on mortality and controlling for both time-static inter-municipal and time-dynamic intra-municipal confounders, we enhanced the precision and accuracy of our estimates [25, 26].

Our findings reveal an exponential risk of mortality after exceeding the optimum mean temperature (MMT = 19ºC), with risk peaking on the exposure day and persisting for up to five days at elevated temperatures. This pattern is qualitatively consistent with previous research in several regions and cities, including the Lisbon metropolitan area, Portugal [45], multiple Mediterranean cities [22], and Hulunbuir in China [46]. However, our region-specific MMT and associated mortality risks at proximate temperatures, differ from a broader European analysis [14], which included data from Alentejo between 1998 and 2012. In this study, a 5–24% risk increase was reported at daily mean temperatures of approximately 26ºC and 29ºC relative to an optimum temperature of approximately 23.5ºC. In contrast, we observed an increase from 8 to 45% at temperatures of 24.7ºC and 28.7ºC. These disparities likely result from our refined, municipality-level, model-driven MMT estimation, contrasting with the broader regional aggregations, which are more prone to biases. The subperiod analyses further support this issue, by excluding disparities resulting from data reflecting temporal shifts in the MMT. The most recent interval (1998–2015) closely aligns with the European analysis, with its specific MMT consistent with that of earlier subperiods (1980–1997) and the overall period. This contrasts with São Paulo, Brazil, where a 1.7ºC rise in MMT and a decrease in heat-related risks were reported between 2000 and 2018 [10]. While no significant correlation was found between MMT and annual mean temperature, some association between risks and annual extreme heat was observed across different population groups. It was hypothesised that both climatic and non-climatic adaptation, such as improved health care coverage and healthier lifestyles, contributed to this trend. In our study, while no long-term decrease in heat sensitivity was observed via changes in the MMT, lower mortality risks in the most recent subperiod suggest some degree of adaptation, though this was not statistically significant. Additional factors potentially contributing to the Alentejo population’s adaptative response might include increased awareness of heat-related health impacts and the rise in residential air conditioning, a key behavioral strategy for reducing heat strain [8–10].

On the other hand, our seasonal analysis does not show any substantial shift in MMT, though risk magnitudes increase in late summer without reaching statistical significance. Another study [47], assuming decadal acclimatisation, reported an increase in heat-related risk and deaths in Prague, Czech Republic, over the last two decades, attributed to both climatic factors and poor adaptation, such as the lack of heat-health warning systems. Considering our results and the upward trajectory in heat-related mortality - approximately in 5,296.4 deaths in Alentejo, between 1980 and 2015 – this may indicate a poor overall adaptation to increasing temperatures and more frequent hot days across the extended summer. The effectiveness of the current early warning system may be contributing to this scenario. In Alentejo, where the population is presumed to have greater heat tolerance (physiological acclimatisation), red alerts are triggered at 35ºC in May-June (vs. 32ºC in other regions), and at 38ºC in July-September (vs. 35ºC). However, our findings did not show evidence of short-term adaptation over the summer, as observed elsewhere [38]. Instead, the higher risks during summer peak suggest a limited capacity for adaptation to more frequent and/or intense heat. Moreover, the number of days with observed-forecasted temperatures excludes the acute effects of single-day exposures and any delayed effects. While exceptional heatwaves, such as the 2003 event that accounted for 35.68% of all deaths in Alentejo, underscore the gravity of prolonged extreme events, the most significant impact of heat often occurs on single hot days or during periods with moderately high, but frequent, temperatures [48]. Such pattern may have implications for the effectiveness of health prevention plans focused on extreme and prolonged temperatures. For example, in Spain, provinces that implemented more actions from their plan saw stronger reductions in mortality attributable to extreme heat, but not to moderate temperatures [49]. Therefore, the current regional assumptions and criteria may be overly aspirational, potentially overlooking significant negative effects. While acknowledging the potential ineffectiveness of frequent warnings due to desensitisation or disregard [50], notifications should be issued in a timely manner, before the onset of critical heat events, rather than on the event itself [51], as practiced in some European countries [52]. Complementary strategies, such as psychoeducation, may play a vital role in raising awareness and preparedness [19] for ongoing and more moderate hazards.

In addition, the specificity of trigger thresholds is critical for enhancing the confidence and efficacy of the prevention systems. This involves greater understanding of the exposure-response curve, which should further be integrated with forecasted and monitored atmospheric indicators [3, 5–7, 19, 20]. While we acknowledge the contribution of the íCARO-alert-index in monitoring potential mortality excess during warmer or heatwave periods, it is important to note that it relies on a linear regression model with a simple lag structure and dynamic maximum temperature thresholds, assuming within-summer population adaptation to heat [18]. These factors may introduce biases and underestimate mortality effects compared to more flexible methods like the DLNM [20, 38, 53] employed in our study.

In our thorough risk evaluation, the daily mean temperature emerged as a practical metric, capturing the impact of two crucial daily thresholds [3]. The reduced thermal amplitude caused by increased minimum temperatures exacerbates heat stress, limiting the body’s ability to recover from daytime exposure. Thus, daily mean temperature might provide a more accurate reflection of overall heat exposure than either maximum or minimum temperatures [48], while also offering improved interpretability for effective decision-making. This study revealed increased risks above the regional optimum temperature across all subregions, although no significant inter-regional variability was observed. While indicating comparable heat vulnerabilities, Alentejo Litoral showed a slightly steeper association slope, which may reflect differential acclimatisation processes. The maritime influence on the climate in this area, where populations in cooler regions often exhibit lower heat tolerance compared to those accustomed to warmer climates [13, 54, 55], could contribute to this pattern. However, the highest risks do not necessarily correspond to the highest net impact, as it depends on the frequency and intensity of nonoptimal exposures. Consequently, Alto Alentejo reported the highest mortality burden, accounting for 3.57% of all deaths in the subregion between 1980 and 2015. Further investigation is warranted to explore heterogeneities related to health aspects (e.g., prevalence of chronic conditions), population characteristics (e.g., age composition, population density), atmospheric-related variables (e.g., humidity levels, wind speed), topographical and environmental factors (e.g., vegetation indexes, urban vs. rural settings), socioeconomic indicators (e.g., income levels, educational attainment, deprivation indices) and infrastructural conditions (e.g., housing quality, air conditioning availability, access to healthcare) [26, 56].

Projections suggest a marked increase in the future burden of heat-related mortality in Alentejo and its subregions over the coming decades. Under the higher emission scenario (RCP8.5), regional heat-related mortality is expected to more than quadruple by the end of the century (2081–2100), reaching a relative excess of 15.88% compared to the 3.64% estimated for the past two decades. Under the lower emissions pathway (RCP4.5), the long-term hazard could be reduced by more than 50%, lowering the relative excess to 6.61%. Similar qualitative findings were reported in a multi-country-city study [53], where hotter climates, such as southern Europe, showed a heightened impact in both scenarios. In contrast to our results, the European study [14] projected a s smaller shift of 4–5% by 2070–2099, relative to 1976–2005, for the Alentejo under the worst-case scenario. These discrepancies likely stem from differences in simulated climatologies, as well as variations in spatial scopes, datasets, and methodologies. While all studies employed the same bias correction method, our approach differed by utilising the outputs of RCMs instead of GCMs. Regional models are expected to add climatic detail, reduce biases and adjust climate change signals of the driving global models [57], hence contributing to the specificity of our analysis. Given the consistent and escalating heat impacts projected across all subregions until 2100 – with both scenarios showing similar magnitudes until 2040 - generalised and immediate implementation of adaptative policies and actions is crucial to mitigate the pervasive effects of heat.

Alongside the major strengths of the current study, there are some limitations that warrant acknowledgment. First, our analysis did not account for effect modifiers or environmental time-varying confounders, like air pollution and humidity. As the effects of climate change become more pronounced, the common co-occurrence of extreme temperature events in conjunction with elevated pollution levels is expected to rise, and meta-analytical evidence suggests that higher levels of PM10 and ozone elevate the heat-related all-cause and non-accidental mortality [58]. Moreover, some studies indicate that dry-hot events may pose a higher risk of mortality than wet-hot events [59], though the synergetic effects remain controversial [60].

Additionally, we did not examine the temperature-mortality associations by levels of particular clinical and demographic factors. One study [15], for example, reported strongest effects related to respiratory and cardiovascular mortality. However, as the implied level of data disaggregation would prevent the provider from releasing the data, we could not disentangle this type of vulnerability. Despite this limitation, we increased the specificity and interpretability of our findings by excluding external causes (e.g., suicides) and mental and behavioural disorders which might have a multifactorial etiology [61, 62], rather than a direct sensitivity to temperature. This exclusion, however, means that our results may not generalize to mental health conditions. Consistent differential susceptibility to heat-induced harm is also found in the elderly population [8, 15, 46, 56, 63]. More than 80% of the included deaths in our dataset occurred among individuals older than 65 years. Therefore, conducting specific analyses for this subgroup may not substantially alter the results. Furthermore, age stratification could have restricted our ability to provide stable and precise estimates. While this is a limitation, the predominance of older adults in our study population underscores their vulnerability to the adverse effects of heat. On the other hand, the ageing trajectory in Alentejo amplifies concerns, as the ageing index for the region has risen from 161.9% in 2001 to 214.9% by 2022 [64]. Such demographic shifts could also exacerbate the projected mortality burden [8, 63, 65, 66]. However, like previous studies [14, 36, 67, 68], we did not account for potential demographic shifts or ongoing adaptations to heat. As a result, our projections may be underestimated in some respects and overestimated in others. Future research could benefit from integrating region-specific population projections and potential acclimatization adjustments with the latest advancements in climate modelling, such as the CMIP6 models featured in IPCC’s AR6 [69]. These models incorporate new emissions and concentration pathways (RCPs), combined with different socioeconomic futures—known as Shared Socioeconomic Pathways (SSPs)—and account for various climate policy-driven mitigation responses throughout the 21st century [2]. Incorporating such updates, especially when extended to regional climate simulations and integrated with global warming thresholds (e.g., + 1.5 °C, + 2.0 °C, + 3.0 °C above pre-industrial levels) [70], could enhance the accuracy of regional projections, and increase confidence in projected changes, while aligning with global policy targets.

Finally, our approach does not account for the inherent uncertainty in the functional trend used to extrapolate exposure-response curves beyond the observed temperatures [36]. This leaves a portion of the epidemiological uncertainty in the projected impacts unaccounted for, adding to the substantial uncertainty arising from the imprecision in observed temperature-mortality associations and climatic projections. This complexity underscores the challenges in accurately assessing the full impact of climate change on heat-related health outcomes. Despite these limitations, our results clear demonstrate the effects of both mitigated and unmitigated scenarios on heat-related premature deaths through the end of the century.

## Conclusion

Heat stress contributes significantly to premature mortality in Alentejo and its subregions, with projections indicating a worsening trend. Without adaptative responses and effective mitigation policies, climate change could amplify the current heat-related mortality rates by more than four times by the end of this century. The potential for substantial reductions in this trajectory under a lower emission scenario highlights the urgency of global actions to mitigate temperature rise. While future studies should explore the variable vulnerabilities among different demographic groups, the present findings clearly signal the need for informed policymaking and the implementation of strong health protection frameworks. Proactive interventions are essential to lessen the widespread impact of heat on Alentejo’s populace. Our research provides critical data to steer such imperative policy and health strategy developments.

## Electronic Supplementary Material

Below is the link to the electronic supplementary material.

Supplementary Material 1

## Data Availability

The observational gridded dataset (Iberia01) providing daily temperatures that was used and analysed during the current study are available in the DIGITAL.CSIC repository (https://digital.csic.es/handle/10261/183071). The EURO-CORDEX RCMs’ temperature data will be provided by the first author upon request. The mortality data that support the findings of this study are available from the first author but restrictions apply to the availability of these data, which were used under license from the Portuguese National Institute of Statistics (INE), Portugal, for the current study, and so are not publicly available. Data are, however, available from the first author upon reasonable request and permission from INE.

## Abbreviations

AF: Attributable Fraction
AN: Attributable Number
AR: Assessment Report
CI: Confidence interval
CMIP: Coupled Model Intercomparison Project
CORDEX: Coordinated Regional Downscaling Experiment
DLNM: Distributed Lag Non-linear Model
ESGF: Earth System Grid Federation
eCI: Empirical Confidence Interval
EURO-CORDEX: European domain of CORDEX
GCM: Global Climate Model
ICD: International Classification of Diseases
IPCC: Intergovernmental Panel on Climate Change
ISI-MIP: Inter-Sectoral Impact Model Intercomparison Project
MedECC: Mediterranean Experts on Climate and Environmental Change
MMT: Minimum Mortality Temperature
RCM: Regional Climate Model
RCPs: Representative Concentration Pathways
RR: Relative Risk
SSPs: Shared Socioeconomic Pathways
