# Supplementary material for: Historical and future heat-related mortality in Portugal’s Alentejo region
Source: BMC Public Health. 2024 Dec 20;24:3552. doi: 10.1186/s12889-024-21058-8 (PMC11662523; doi:10.1186/s12889-024-21058-8)
Supplement: Supplementary file 1 — Supplementary Material 1 [file 12889_2024_21058_MOESM1_ESM.docx]

Supplemental Material

**Historical and future heat-related mortality in Portugal’s Alentejo region**

# **Statistical modelling framework**

The case time-series design [1-2] was used to model series of daily mean temperature and mortality count data at the municipality level (i.e., cases) in Alentejo. The data was fitted using a conditional Poisson regression model with Poisson distribution and fixed-effects estimators [2-3]. The expected counts of deaths E(𝑦*_md_*) at day *d* (from 1 to the number of days) in municipality *m* (*m* = 1, …, 47) were modelled as follows [2, 4]:

*g*[E(*y_md_*)] = ξ*_m(k)_* + *f* (*x_md_, l; ϴ*) + *s(d; β) + I(w_d_)*

Here, *g* is the logarithm link function, and *ξ_m(k_)* represents stratum-specific intercepts, conditioned out as nuisance parameters, with each stratum *k* defined by unique combinations of municipality, year, and month of the seasonal period. These parameters capture within-case variations in baseline mortality risk, while broader temporal trends shared across the entire territory are captured by two additional terms: *s(d)*, a natural cubic spline of the seasonal day with three degrees of freedom (df) interacting with year indicators (1980, …, 2015), adjusted for non-constant seasonal over time; and *I(W_d_),* indicator variables for each day of the week *w* (*w* = 1,… 7), capturing day-specific mortality fluctuations^2^.

The short-term association between mortality and temperature *x_md_* was characterised using a distributed lag non-linear model (DLNM), fitted through a *cross-basis* function *f* (*x_md_, l*), to fit both the exposure-response and lag-response relationships, accounting for nonlinear and delayed effects [5, 6]. In line with common practices in time-series analyses [2, 5], this bi-dimensional parameterization was defined using natural cubic splines. The temperature-mortality dimension used two internal knots from the Alentejo´s temperature distributions (50th and 90th percentiles). The lag-response dimension, modelled over a lag window *l* = 0, …, 10, used two internal knots at equally spaced values on a log scale to account for any delayed exposure effects and potential short-term mortality displacement [7-8].

**Table S1.** Selected EURO-CORDEX RCMs used for the historical, RCP4.5, and RCP8.5 experiments, along with their respective forcing GCMs and responsible institutes.

| **RCM name**  (Version ID, Ensemble Member) | **GCM name** | **Institute** |
| --- | --- | --- |
| ALADIN63  (v2, r1i1p1) | CNRM-CERFACS-CNRM-CM5 | Météo-France/Centre National de Recherches Météorologiques (CNRM), France |
| HIRHAM5  (v2, r3i1p1) | ICHEC-EC-Earth | Danmarks Meteorologiske Insitut (DMI), Denmark |
| RACMO22E  (v1, r12i1p1) | ICHEC-EC-Earth | Koninklijk Nederlands Meteorologisch Instituut (KNMI), Netherlands |
| REMO2009  (v1, r1i1p1) | MPI-M-MPI-ESM-LR | Max-Planck-Institut für Meteorologie (MPI-M) and Climate Service Center (CSC), Germany |
| REMO2015  (v1, r1i1p1) | NCC-NorESM1-M | Climate Service Center Germany (GERICS), Germany |

**Table S2.** Heat-related cumulative relative risks over 0-10 days associated with various daily mean temperature thresholds (regional percentiles) over the time subperiods 1980-1997 and 1998-2015. Effect estimates are presented for both scenarios, including and excluding the years with exceptional mortality rates, with regard to the specific subperiod reference temperature (MMT = 19ºC).

| **Temperature**  (Percentile) | **Cumulative relative risk (95% CI)** | | **z-statistic** | ***p*-value*** |
| --- | --- | --- | --- | --- |
|  | 1980-1997 | 1998-2015 |  |  |
| Including the whole years | | | | |
| 22.1 (P50) | 0.99 (0.96, 1.03) | 1.04 (1.00, 1.07) | -1.163 | 0.245 |
| 24.7 (P75) | 1.03 (0.95, 1.11) | 1.12 (1.06, 1.20) | -1.274 | 0.203 |
| 28.7 (P95) | 1.41 (1.28, 1.56) | 1.47(1.35, 1.60) | -0.412 | 0.680 |
| 31.2 (P99) | 2.11 (1.82, 2.45) | 1.92 (1.69, 2.17) | 0.681 | 0.496 |
| Excluding years of 1981, 1991, 2003, 2006 and 2010 | | |  |  |
| 22.1 (P50) | 0.99 (0.95, 1.03) | 1.03 (0.99, 1.07) | -1.149 | 0.251 |
| 24.7 (P75) | 1.02 (0.94, 1.11) | 1.11 (1.03, 1.20) | -1.022 | 0.307 |
| 28.7 (P95) | 1.41 (1.27, 1.56) | 1.34 (1.21, 1.48) | 0.500 | 0.617 |
| 31.2 (P99) | 2.10 (1.78, 2.46) | 1.58 (1.32, 1.88) | 1.642 | 0.101 |

**p*-values for the statistical difference between the two time periods (Z-test, significance level < 0.05).

**Table S3.** Heat-related cumulative relative risks over 0-10 days associated with various daily mean temperature thresholds (regional percentiles) over the early (May-June) and late (July-September) warm season. Effect estimates are presented for both scenarios, including and excluding the years with exceptional mortality rates, with regard to the specific subperiod reference temperature (MMT = 19ºC).

| **Temperature**  (Percentil) | **Cumulative relative risk (95% CI)** | | **z-statistic** | ***p*-value*** |
| --- | --- | --- | --- | --- |
|  | May-June | July-September |  |  |
| Including the whole years | | | | |
| 22.1 (P50) | 1.06 (1.00, 1.14) | 1.10 (1.02, 1.18) | -0.432 | 0.665 |
| 24.7 (P75) | 1.16 (1.04, 1.30) | 1.19 (1.09, 1.30) | -0.218 | 0.828 |
| 28.7 (P95) | 1.31 (1.10, 1.57) | 1.55 (1.40, 1.72) | -1.130 | 0.258 |
| 31.2 (P99) | 1.40 (1.00, 1.95) | 2.21 (1.92, 2.55) | -1.748 | 0.080 |
| Excluding years of 1981, 1991, 2003, 2006 and 2010 | | |  |  |
| 22.1 (P50) | 1.03 (0.94, 1.13) | 1.06 (0.94, 1.19) | -0.271 | 0.786 |
| 24.7 (P75) | 1.07 (0.91, 1.26) | 1.15 (0.99, 1.32) | -0.434 | 0.664 |
| 28.7 (P95) | 1.23 (0.94, 1.61) | 1.41 (1.19, 1.67) | -0.608 | 0.543 |
| 31.2 (P99) | 1.40 (0.80, 2.43) | 1.71 (1.29, 2.26) | -0.452 | 0.651 |

**p*-values for the statistical difference between the two time periods (Z-test, significance level < 0.05).

**Table S4.** Overall absolute (prevalence) and relative (%) heat-related excess mortality for Alentejo and subregions during the 1980 to 2015 period.

| **Subregion/Region** | **Excess mortality (1980-2015**) | |
| --- | --- | --- |
|  | Absolute  [prevalence (95% eCI)] | Relative  [% (95% eCI)] |
| Alentejo Central | 987.85 (163.56, 2055.52) | 1.51 (0.33, 3.14) |
| Alentejo Litoral | 607.71 (35.82, 1175.38) | 1.59 (0.23, 3.21) |
| Alto Alentejo | 2114.44 (1227.47, 3006.92) | 3.57 (1.88, 5.02) |
| Baixo Alentejo | 1285.56 (23.58, 2522.81) | 1.98 (0.14, 5.02) |
| Alentejo | 5296.4 (3435.72, 7211.27) | 2.32 (1.42, 3.19) |

**Table S5.** Projected overall absolute (prevalence) and relative (%) heat-related excess mortality for Alentejo and subregions up to 2100.

| **Region** | **RCP** | **Period** | **Excess mortality (1981-2100)** | |
| --- | --- | --- | --- | --- |
|  |  |  | Absolute  [prevalence (95% eCI)] | Relative  [% (95% eCI)] |
| Alentejo Central | RCP45 | 1981 - 2000 | 458.55 (0.60 – 1,071.79) | 3.43 (0.00 - 8.02) |
|  |  | 2001 - 2020 | 585.07 (15.88 – 1,255.92) | 4.38 (0.12 - 9.39) |
|  |  | 2021 - 2040 | 705.26 (17.14 – 1,443.85) | 5.28 (0.13 - 10.8) |
|  |  | 2041 - 2060 | 865.03 (40.88 – 1,697.09) | 6.47 (0.31 - 12.69) |
|  |  | 2061 - 2080 | 1,028.78 (75.45 – 1,890.18) | 7.70 (0.56 - 14.14) |
|  |  | 2081 - 2100 | 1,068.12 (79.73 – 2,020) | 7.99 (0.60 - 15.11) |
|  | RCP85 | 1981 - 2000 | 461.01 (2.01 – 1,081.26) | 3.45 (0.02 - 8.09) |
|  |  | 2001 - 2020 | 561.47 (1.81 – 1,235.49) | 4.20 (0.01 - 9.24) |
|  |  | 2021 - 2040 | 787.22 (7.63 – 1,561.16) | 5.89 (0.06 - 11.68) |
|  |  | 2041 - 2060 | 1,068.12 (93.22 – 1,919.9) | 7.99 (0.70 - 14.36) |
|  |  | 2061 - 2080 | 1,639.37 (304.53 – 2,642.99) | 12.26 (2.28 - 19.77) |
|  |  | 2081 - 2100 | 2,332.75 (744.49 – 3,412.71) | 17.45 (5.57 - 25.53) |
| Alentejo Litoral | RCP45 | 1981 - 2000 | 118.68 (-282.56 - 494.79) | 1.52 (-3.61 - 6.33) |
|  |  | 2001 - 2020 | 214.22 (-183.34 - 633.62) | 2.74 (-2.34 - 8.10) |
|  |  | 2021 - 2040 | 297.87 (-118.02 - 777.04) | 3.81 (-1.51 - 9.94) |
|  |  | 2041 - 2060 | 454.31 (2.41 – 1,021.91) | 5.81 (0.03 - 13.07) |
|  |  | 2061 - 2080 | 548.17 (27.59 – 1,141.07) | 7.01 (0.35 - 14.59) |
|  |  | 2081 - 2100 | 598.44 (34.96 – 1,251.53) | 7.65 (0.45 - 16.01) |
|  | RCP85 | 1981 - 2000 | 116.97 (-284.74 - 493.09) | 1.5 (-3.64 - 6.31) |
|  |  | 2001 - 2020 | 210.10 (-175.41 - 628.07) | 2.69 (-2.24 - 8.03) |
|  |  | 2021 - 2040 | 372.71 (-46.22 - 893.9) | 4.77 (-0.59 - 11.43) |
|  |  | 2041 - 2060 | 584.83 (40.59 – 1,185.18) | 7.48 (0.52 - 15.16) |
|  |  | 2061 - 2080 | 988.11 (158.98 – 1,721.84) | 12.64 (2.03 - 22.02) |
|  |  | 2081 - 2100 | 1,495.16 (437.22 – 2,362.37) | 19.12 (5.59 - 30.22) |
| Alto Alentejo | RCP45 | 1981 - 2000 | 823.19 (90.40 – 1,403.34) | 6.83 (0.75 - 11.64) |
|  |  | 2001 - 2020 | 946.32 (165.17 – 1,569.03) | 7.85 (1.37 - 13.01) |
|  |  | 2021 - 2040 | 1,056.53 (199.15 – 1,730.4) | 8.76 (1.65 - 14.35) |
|  |  | 2041 - 2060 | 1,193.90 (220.45 – 1,942.5) | 9.90 (1.83 - 16.11) |
|  |  | 2061 - 2080 | 1,373.57 (353.97 – 2,112.67) | 11.39 (2.94 - 17.52) |
|  |  | 2081 - 2100 | 1,407.26 (392.86 – 2,254.58) | 11.67 (3.26 - 18.69) |
|  | RCP85 | 1981 - 2000 | 817.50 (91.86 - 1390.29) | 6.78 (0.76 - 11.53) |
|  |  | 2001 - 2020 | 940.01 (131.37 – 1,568.11) | 7.79 (1.09 - 13.00) |
|  |  | 2021 - 2040 | 1,095.89 (161.01 – 1,776.14) | 9.09 (1.34 - 14.73) |
|  |  | 2041 - 2060 | 1,352.37 (350.25 – 2,110.42) | 11.21 (2.90 - 17.5) |
|  |  | 2061 - 2080 | 1,786.10 (654.59 - 2618) | 14.81 (5.43 - 21.71) |
|  |  | 2081 - 2100 | 2,340.26 (1,194 – 3,187.65) | 19.41 (9.90 - 26.43) |
| Baixo Alentejo | RCP45 | 1981 - 2000 | 560.68 (16.79 – 1,186.99) | 4.23 (0.13 - 8.96) |
|  |  | 2001 - 2020 | 718.63 (84.22 – 1,411.84) | 5.42 (0.64 - 10.65) |
|  |  | 2021 - 2040 | 858.90 (110.69 – 1,633.78) | 6.48 (0.84 - 12.33) |
|  |  | 2041 - 2060 | 1,060.84 (173.58 – 1,937.05) | 8 (1.31 - 14.61) |
|  |  | 2061 - 2080 | 1,246.13 (313.41 – 2,131.49) | 9.4 (2.36 - 16.08) |
|  |  | 2081 - 2100 | 1,292.78 (303.2 – 2,251.13) | 9.75 (2.29 - 16.98) |
|  | RCP85 | 1981 - 2000 | 568.15 (22.16 – 1,195.92) | 4.29 (0.17 - 9.02) |
|  |  | 2001 - 2020 | 692.00 (52.59 – 1,395.82) | 5.22 (0.40 - 10.53) |
|  |  | 2021 - 2040 | 965.06 (106.51 – 1,792.53) | 7.28 (0.80 - 13.52) |
|  |  | 2041 - 2060 | 1,293.34 (308.33 – 2,180.52) | 9.76 (2.33 - 16.45) |
|  |  | 2061 - 2080 | 1,940.97 (754.92 – 2,918.2) | 14.64 (5.70 - 22.02) |
|  |  | 2081 - 2100 | 2,724.85 (1537.43 – 3,722.9) | 20.56 (11.6 - 28.09) |
| Alentejo | RCP45 | 1981 - 2000 | 1,322.51 (51.72 – 2,953.87) | 2.84 (0.11 - 6.35) |
|  |  | 2001 - 2020 | 1,751.98 (200.23 – 3,616.72) | 3.77 (0.43 - 7.78) |
|  |  | 2021 - 2040 | 2,050.77 (265.41 – 4,189.55) | 4.41 (0.57 - 9.01) |
|  |  | 2041 - 2060 | 2,561.31 (358.44 – 4,983.26) | 5.51 (0.77 - 10.72) |
|  |  | 2061 - 2080 | 3,167.40 (757.72 – 5,737.92) | 6.81 (1.63 - 12.34) |
|  |  | 2081 - 2100 | 3,075.62 (767.74 – 5,733.26) | 6.61 (1.65 - 12.33) |
|  | RCP85 | 1981 - 2000 | 1,334.07 (56.17 – 2,944.81) | 2.87 (0.12 - 6.33) |
|  |  | 2001 - 2020 | 1,691.85 (144.91 – 3,539.68) | 3.64 (0.31 - 7.61) |
|  |  | 2021 - 2040 | 2,246.15 (353.79 – 4,493.35) | 4.83 (0.76 - 9.66) |
|  |  | 2041 - 2060 | 3,173.94 (771.39 – 5,779.55) | 6.83 (1.66 - 12.43) |
|  |  | 2061 - 2080 | 4,953.10 (1,962.96 – 8,177.67) | 10.65 (4.22 - 17.59) |
|  |  | 2081 - 2100 | 7,384.14 (3,814.61 – 10,692.74) | 15.88 (8.20 - 22.99) |

**
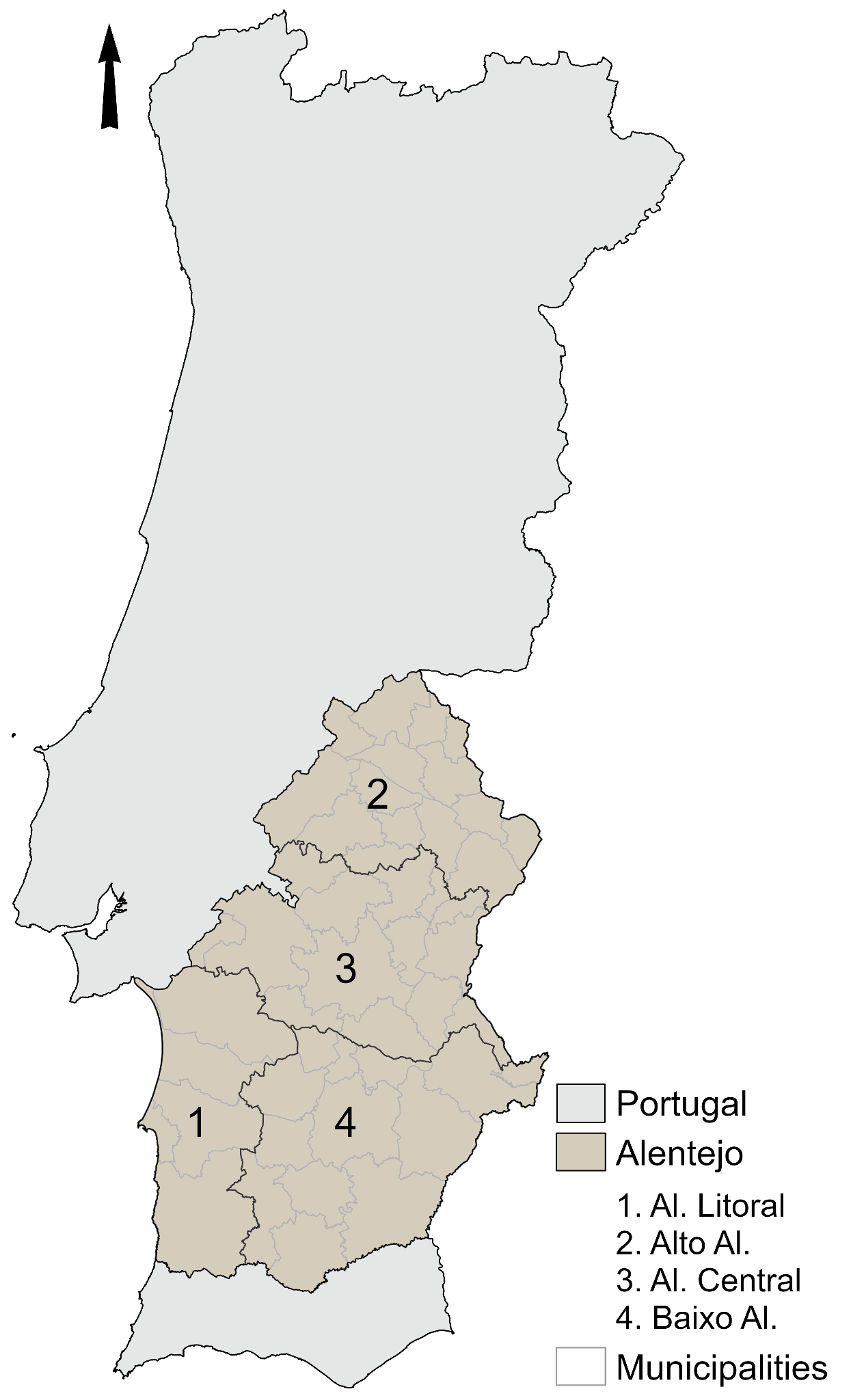
**

**Figure S1.** Geographical context of the study area. Al.= Alentejo


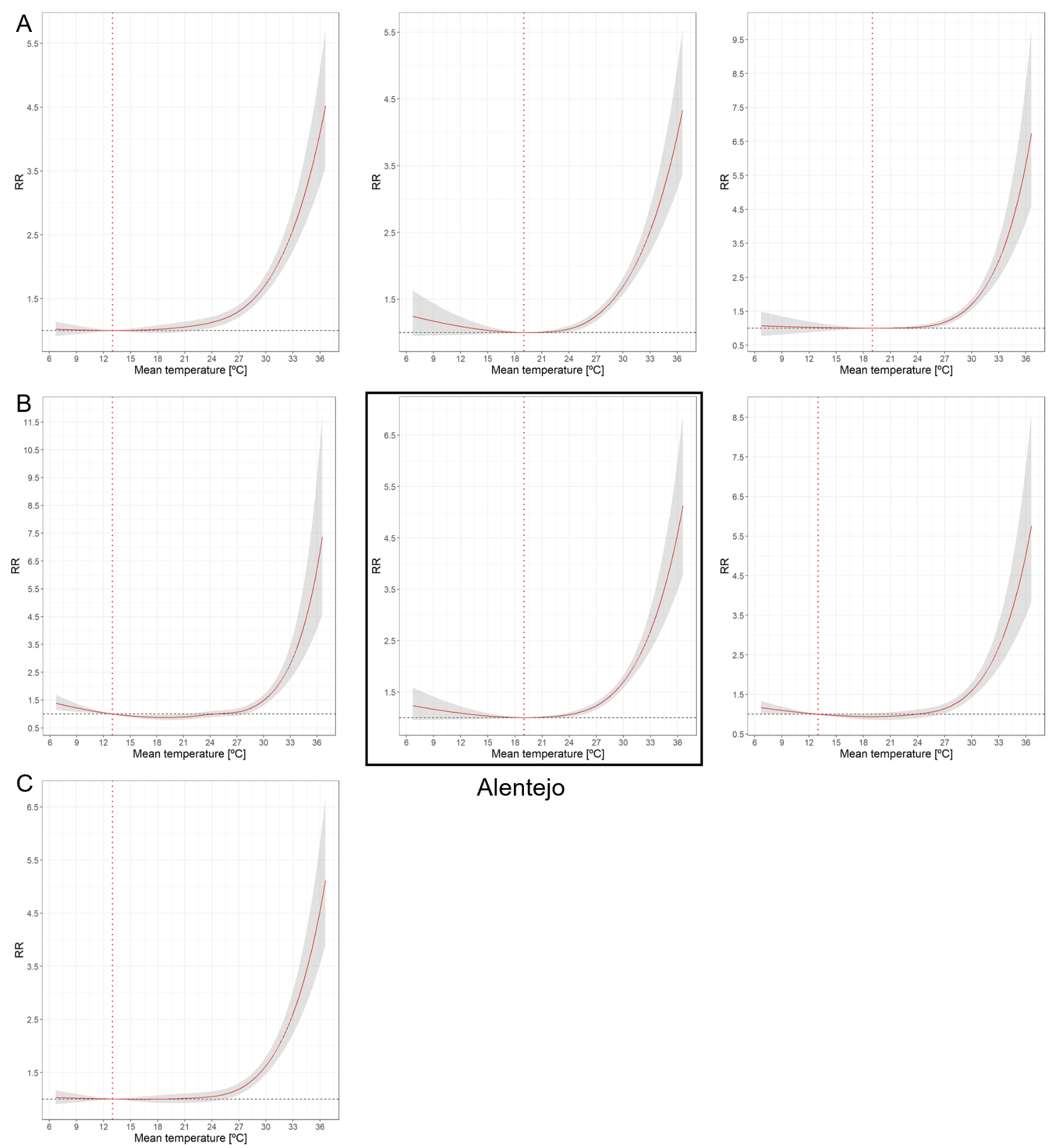


**Figure S2.** Sensitivity analysis performed for the seasonal temperature-morality association in Alentejo (1980-2015). Original setting of the final model displayed in the center frame. (A) Overall cumulative relative risk (RR) using various combinations of percentiles for knot placement across the daily mean temperature range: 50th, 75th, and 90th; 50th and 75th; and 75th and 90th. (B): RR using different maximum lag lengths in the lag-response function: 5 days, 10 days (original setting), and 14 days. (C) RR broadening the stratum by excluding the monthly component. Minimum mortality temperature (MMT) for each specific adjustment is represented by the vertical dashed line.


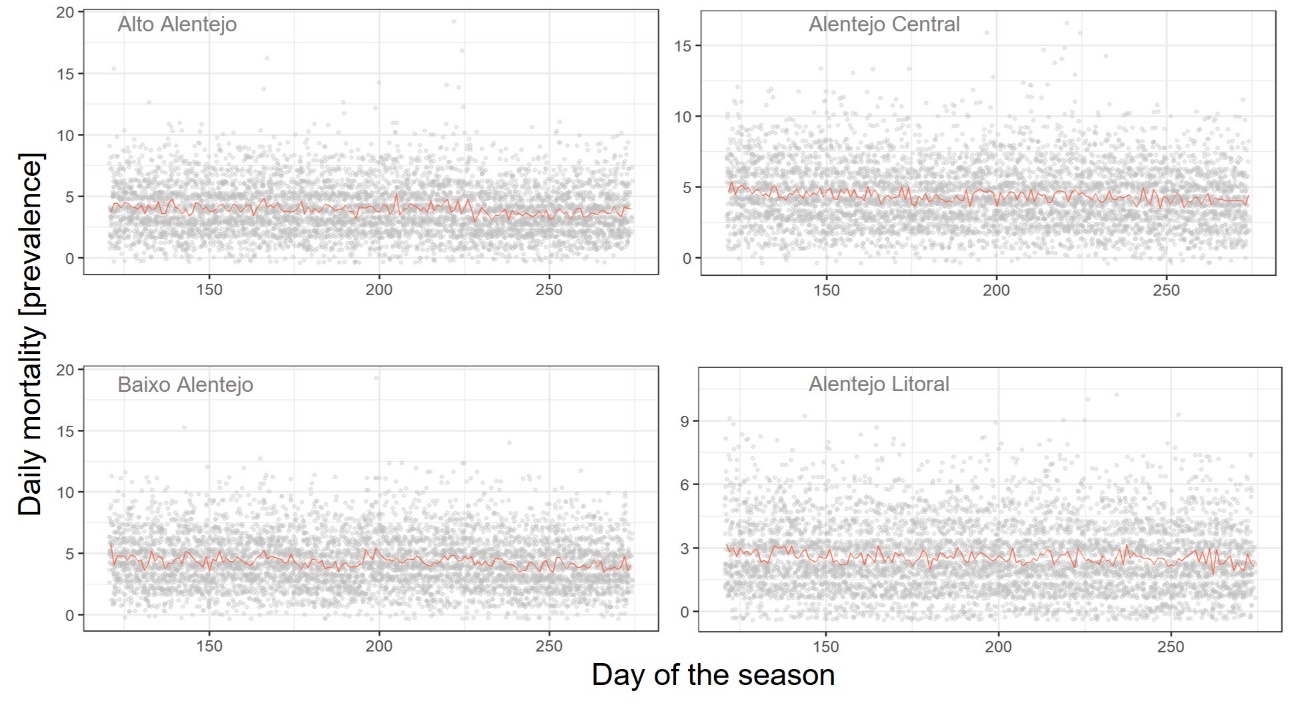


**Figure S3.** Seasonal distribution (May-September) of observed daily mortality in Alentejo’s subregions from 1980 to 2015. The orange line indicates the average daily deaths during this period.

**
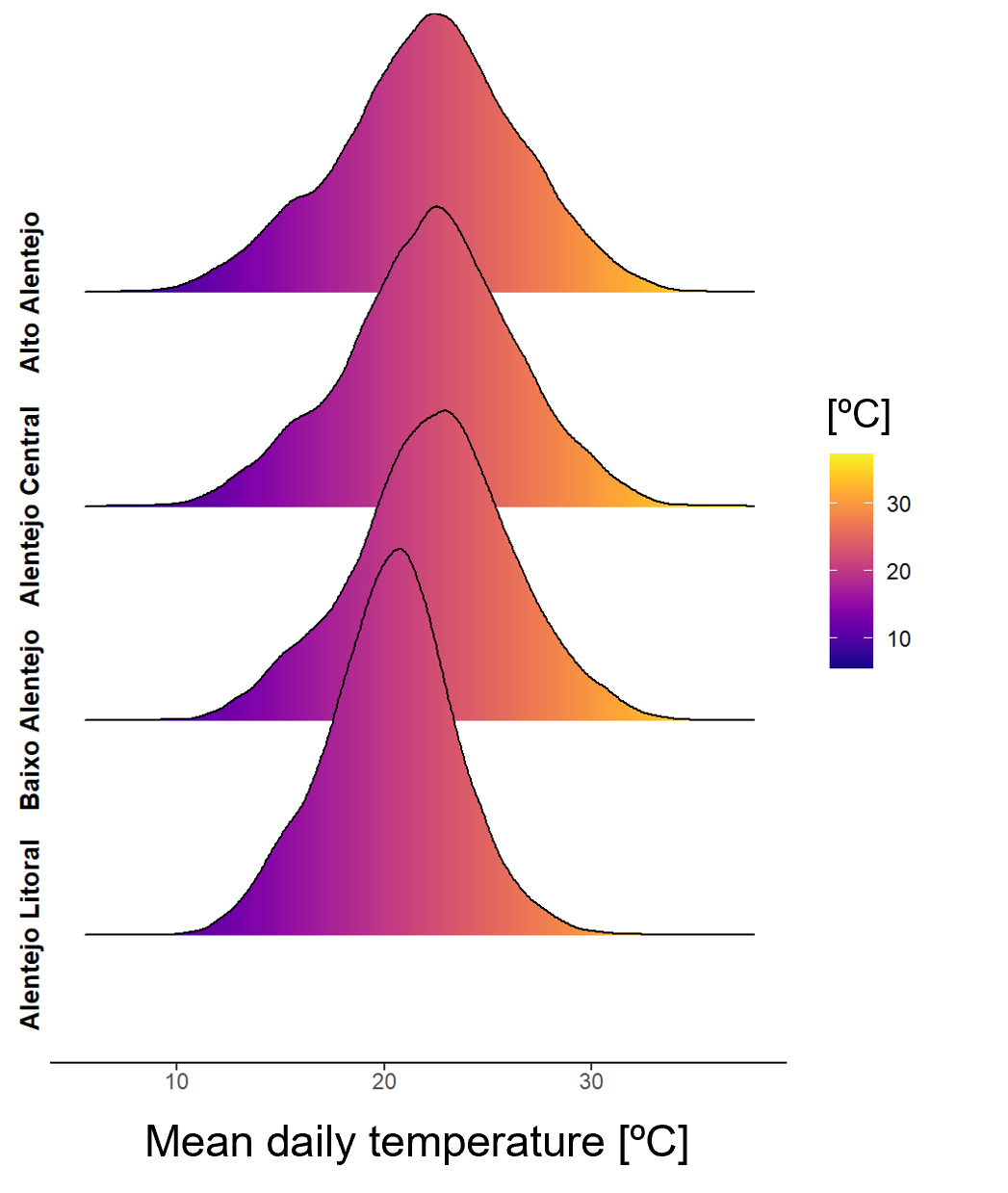
**

**Figure S4.** Seasonal distribution (May-September) of the mean daily temperature in Alentejo’s subregions during the 1980 to 2015 period.


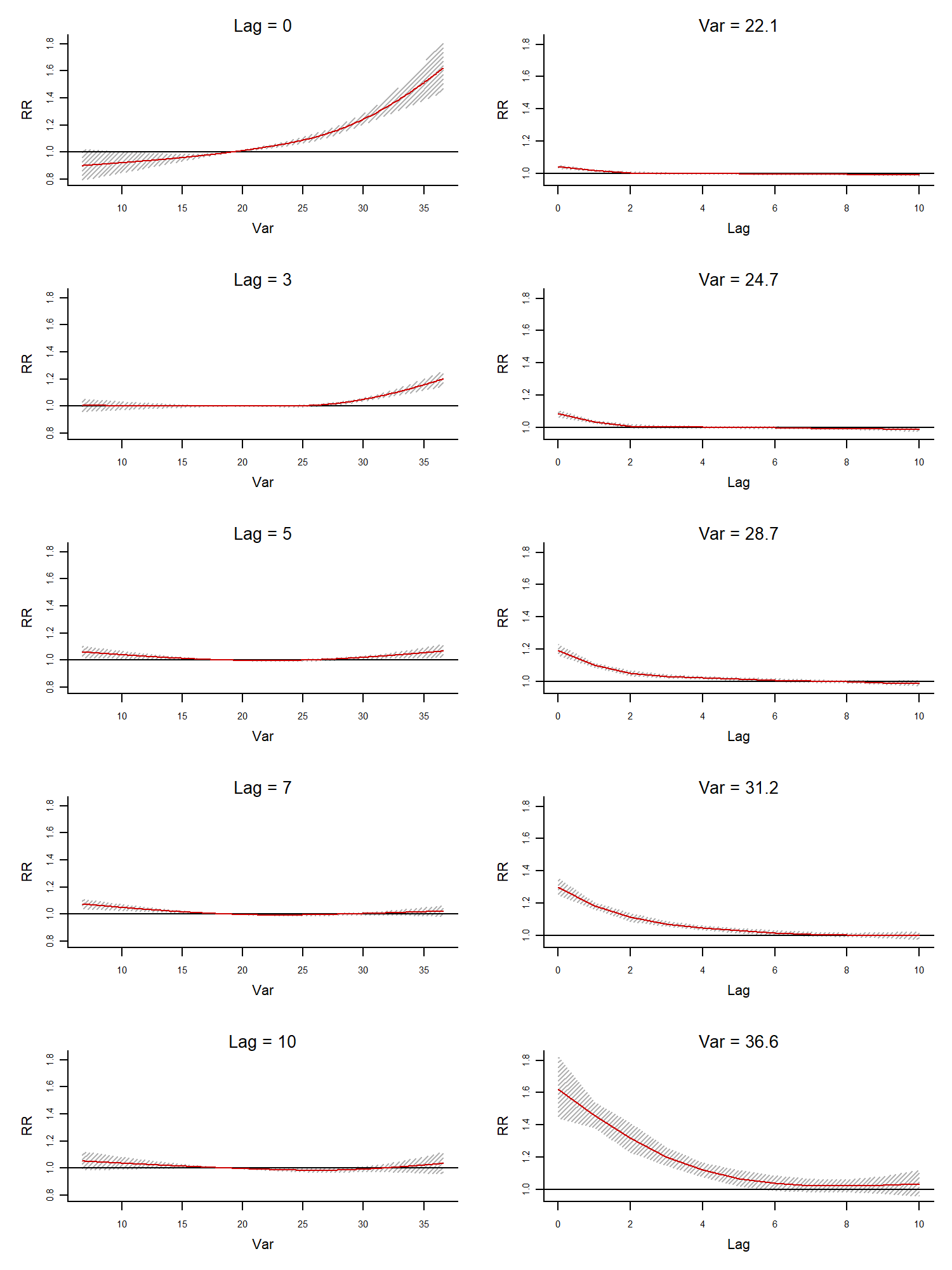


**Figure S5.** Association between daily mean temperature (Var [ºC]) and mortality for Alentejo (1980-2015). Left panel: patterns of the temperature-specific relative risk (RR) at different lags (Lag = 0, day of a given exposure; Lag = 10, 10 days after a given exposure). Right panel: patterns of lag-specific RR at 50^th^ (22.1°C), 75^th^ (24.7°C), 95^th^ (28.7°C), 99^th^ (31.2°C) and maximum extreme (36.6°C) of the temperature distribution. Effects were estimated using the MMT (19.0°C) as reference. Shaded areas represent 95% confidence intervals.

# **References**

1. Gasparrini A. The case time series design. Epidemiology. 2021 Jul 30;32(6):829-837.

2. Gasparrini A. A tutorial on the case time series design for small-area analysis. BMC Med Res Methodol. 2022 Apr 30;22(1).

3. Armstrong BG, Gasparrini A, Tobias A. Conditional poisson models: a flexible alternative to conditional logistic case cross-over analysis. BMC Med Res Methodol. 2014 Nov 24;14(1).

4. Gasparrini A, Masselot P, Scortichini M, Schneider R, Mistry MN, Sera F, et al. Small-area assessment of temperature-related mortality risks in England and Wales: a case time series analysis. The Lancet Planetary Health. 2022 Jul;6(7):e557–e564.

5. Gasparrini A, Armstrong B, Kenward MG. Distributed lag non-linear models. Stat Med. 2010 Aug 26;29(21):2224-2234

6. Gasparrini A. Modelling lagged associations in environmental timesSeries data. Epidemiology. 2016 Nov;27(6):835-842.

7. Tobías A, Armstrong B, Gasparrini A. Brief report: investigating uncertainty in the minimum mortality temperature: methods and application to 52 Spanish cities. Epidemiology. 2017 Jan;28(1):72-76.

8. Gasparrini A, Guo Y, Hashizume M, Lavigne E, Tobias A, Zanobetti A, et al. Changes in susceptibility to heat during the summer: a multicountry analysis. Am J Epidemiol. 2016 May 2;183(11):1027-1036.
